# Effect of sequencing platforms on the sensitivity of chemical mutation detection using Hawk-Seq™

**DOI:** 10.1186/s41021-024-00313-9

**Published:** 2024-10-09

**Authors:** Sayaka Hosoi, Takako Hirose, Shoji Matsumura, Yuki Otsubo, Kazutoshi Saito, Masaaki Miyazawa, Takayoshi Suzuki, Kenichi Masumura, Kei-ichi Sugiyama

**Affiliations:** 1 R&D - Safety Science Research, Kao Corporation, 3-25-14 Tonomachi, Kawasaki-ku, Kawasaki-shi, Kanagawa 210-0821 Japan; 2 R&D - Safety Science Research, Kao Corporation, 2606 Akabane, Ichikai-Machi, Haga-Gun, Tochigi 321-3497 Japan; 3 Division of Genome Safety Science, National Institute of Health Sciences, 3-25-26 Tonomachi, Kawasaki-ku, Kawasaki-shi, Kanagawa 210-9501 Japan; 4 Division of Risk Assessment, National Institute of Health Sciences, 3-25-26 Tonomachi, Kawasaki-ku, Kawasaki-shi, Kanagawa 210-9501 Japan

**Keywords:** Next generation sequencing, Error-corrected sequencing, Mutagenicity assay, Mutational signatures

## Abstract

**Background:**

Error-corrected next-generation sequencing (ecNGS) technologies have enabled the direct evaluation of genome-wide mutations after exposure to mutagens. Previously, we reported an ecNGS methodology, Hawk-Seq™, and demonstrated its utility in evaluating mutagenicity. The evaluation of technical transferability is essential to further evaluate the reliability of ecNGS-based assays. However, cutting-edge sequencing platforms are continually evolving, which can affect the sensitivity of ecNGS. Therefore, the effect of differences in sequencing instruments on mutation data quality should be evaluated.

**Results:**

We assessed the performance of four sequencing platforms (HiSeq2500, NovaSeq6000, NextSeq2000, and DNBSEQ-G400) with the Hawk-Seq™ protocol for mutagenicity evaluation using DNA samples from mouse bone marrow exposed to benzo[*a*]pyrene (BP). The overall mutation (OM) frequencies per 10^6^ bp in vehicle-treated samples were 0.22, 0.36, 0.46, and 0.26 for HiSeq2500, NovaSeq6000, NextSeq2000, and DNBSEQ-G400, respectively. The OM frequency of NextSeq2000 was significantly higher than that of HiSeq2500, suggesting the difference to be based on the platform. The relatively higher value in NextSeq2000 was a consequence of the G:C to C:G mutations in NextSeq2000 data (0.67 per 10^6^ G:C bp), which was higher than the mean of the four platforms by a ca. of 0.25 per 10^6^ G:C bp. A clear dose-dependent increase in G:C to T:A mutation frequencies was observed in all four sequencing platforms after BP exposure. The cosine similarity values of the 96-dimensional trinucleotide mutation patterns between HiSeq and the three other platforms were 0.93, 0.95, and 0.92 for NovaSeq, NextSeq, and DNBSeq, respectively. These results suggest that all platforms can provide equivalent data that reflect the characteristics of the mutagens.

**Conclusions:**

All platforms sensitively detected mutagen-induced mutations using the Hawk-Seq™ analysis. The substitution types and frequencies of the background errors differed depending on the platform. The effects of sequencing platforms on mutagenicity evaluation should be assessed before experimentation.

**Supplementary Information:**

The online version contains supplementary material available at 10.1186/s41021-024-00313-9.

## Background

Error-corrected next-generation sequencing (ecNGS) technologies have enabled the direct evaluation of genome-wide mutations after exposition to mutagens [1, 2]. ecNGSs were first reported in the early 2010s. They can dramatically reduce the error frequency in next-generation sequencing (NGS) by utilizing complementary strand information, enabling the direct detection of mutagen-induced mutations [3–5]. We reported an ecNGS methodology, Hawk-Seq™, and indicated its utility in obtaining mutation data that reflected mutagenic mechanisms [6]. These ecNGS methods can clarify mutagen-induced genomic mutations with sufficient resolution to identify features of mutagens [7].

The utility of ecNGS methodologies has been demonstrated in several studies [8–11]. However, to further evaluate the reliability of ecNGS-based mutagenicity assays, they need to be evaluated considering diverse characteristics, such as sensitivity, specificity, and reproducibility [12]. According to the OECD Guidance Document on Good In Vitro Method Practices (GIVIMP), to confirm the robustness and reliability of an assay, successful transfer to a range of equipment and different laboratories should be demonstrated [13]. However, several precision instruments are required during ecNGS library construction (e.g., sequencing platforms) and are continuously renewed following technological innovation. The effect of instrument differences on mutation data quality should be evaluated prior to technical transfer because they would disturb the evaluation of within- and between-laboratory reproducibility.

In particular, the background error frequencies are the most critical parameters in ecNGS analysis because their variations can directly decrease the detection sensitivity and data resolution. One of the instruments that would most affect the error frequency is the sequencer because error frequencies have been reported to vary according to the model in several studies [14–16]. Although several sequencing platforms have been used in ecNGS research, direct comparisons among these platforms have not yet been performed. To interpret the transferability of ecNGSs correctly, it is essential to confirm whether differences in sequencing platforms affect ecNGS analysis.

Thus, we evaluated the performance of four commonly used sequencing platforms (HiSeq2500, NovaSeq6000, NextSeq2000, and DNBSEQ-G400) with the Hawk-Seq™ protocol for mutagenicity evaluation using mouse DNA samples exposed to benzo[*a*]pyrene (BP). By evaluating the mutation data produced by each sequencing platform in terms of the error frequency, mutation pattern, and context, we confirmed whether the currently available sequencing platforms could provide equivalent high-resolution mutation data.

## Materials and methods

### Library preparation and sequencing

DNA samples from the bone marrow of male C57BL/6JJmsSlc-Tg (*gpt* delta) mice (7–9 weeks old) prepared in our previous study were used. Briefly, mice were orally administered olive oil or 150 and 300 mg/kg BP (CASRN. 50-32-8) once daily for five days; then, DNA was extracted seven days after the final treatment (Table 1) [6]. DNA samples were sheared into fragments with a peak size of 350 bp using a sonicator (Covaris, MA, USA). The resulting DNA fragments were used for sequence library preparation using the TruSeq Nano DNA Low Throughput Library Prep Kit (TruSeq; Illumina, San Diego, CA, USA), with a slight modification for Hawk-Seq™ [6]. Briefly, the sonicated DNA fragments were subjected to end repair, 3ʹ dA-tailing, and ligation to TruSeq-indexed adaptors, according to the manufacturer’s instructions. The DNA concentration of each ligated sample was measured using the Agilent 4200 Tape Station (Agilent Technologies, CA, USA). The ligated products were diluted to 3.1 pmol/µL with a suspension buffer. Then, 25 µL of the diluted products were subjected to PCR amplification to prepare the sequencing libraries. Illumina libraries were converted to DNBSeq libraries using the MGIEasy Universal library Conversion Kit (MGI, Shenzhen, China). The libraries obtained were sequenced at 2 × 150 or 2 × 151 bp using NovaSeq6000 (NovaSeq; Illumina, San Diego, CA, USA), NextSeq2000 (NextSeq; Illumina), and DNBSEQ-G400 (DNBSeq; MGI Tech Co., Ltd., Shenzhen, China) to yield at least 50 M paired-end reads. The sequence data of HiSeq2500 were obtained as 2 × 100 bp in our previous study [6]. Sequencing experiments were performed at Kao Corporation for NextSeq, and as a service of Takara Bio Inc. and FASMAC Co., Ltd. for the NovaSeq and DNBSeq datasets, respectively.

**Table 1:** Summary of the *gpt* assay and mutation frequency of mouse DNA samples

Sample ID	Dose (mg/kg/day)	Mutant frequency (×10^− 6^) in *gpt* assay	Overall mutation frequency (×10^− 6^ bp) in Hawk-Seq™ analysis			
			HiSeq*	NovaSeq	NextSeq	DNBSeq
1001	0 (Olive oil)	1.34	0.22	0.32	0.43	0.32
1002	0 (Olive oil)	1.49	0.23	0.40	0.50	0.21
1201	150	13.26	0.75	0.85	0.95	0.81
1301	300	26.73	1.24	1.32	1.46	1.38

*: Data from a previous study [6]

### Processing of sequencing data

Adaptor sequences and low-quality bases were removed from the generated paired-end reads using Cutadapt-3.5 [17]. Edited paired-end reads were mapped to the GRCm38 mouse reference genome sequence using Bowtie2-2.4.1 [18]. SAM format processing was performed using SAMtools-1.10 [19]. To prepare the double-stranded DNA consensus sequence (dsDCS), read pairs that shared the same genomic positions (that is, start and end positions on the reference genome) were grouped into same position groups (SP-Gs) and divided into two subgroups based on their R1 and R2 orientations. SP-Gs that included read pairs in both orientations were identified and used to generate dsDCS read pairs [6]. The resulting dsDCS read pairs were mapped to the reference genome sequence using Bowtie2-2.4.1. The obtained SAM files were processed using SAM tools, and mutations were detected.

### Mutation detection and evaluation of similarity of mutation spectrum

To calculate the mutation frequency, the number of base substitutions of each type was enumerated separately. The frequency of each substitution type per 10^6^ G:C or A:T base pairs was calculated by dividing each mutation count by the total dsDCS read base count mapped to the G:C or A:T base pairs, respectively. To reduce background mutation calls, such as single nucleotide polymorphisms (SNPs), the genomic positions listed in the ensemble variation list (version 92) were removed from the analysis [20]. Additionally, the suspected variant positions of *gpt* delta mice observed in our laboratory historical data were removed from the analysis [6]. To determine the 96-dimensional mutation patterns, the bases flanking the 5′ and 3′ of each mutated residue were analyzed. Mutation frequencies were calculated for each trinucleotide. Similarities in the mutation spectra between sequencers were evaluated based on cosine similarity (CS) [21, 22]. Decomposition of trinucleotide signatures to single base substitution signatures (SBSs) were conducted using the deconstructSigs package [23].

## Results

### Sequence output and amount of dsDNA consensus sequence per sample

Sequencing libraries prepared using DNA samples from male *gpt* delta mice exposed to olive oil (Sample ID 1001 and 1002) or 150 (Sample ID 1201) and 300 mg/kg BP (Sample ID 1301) were subjected to sequencing using four platforms. At least 50 M read pairs were obtained for each library on each platform (Table 2). In all the data, more than 90% of the read bases indicated a quality score of 30 or higher. After data processing for Hawk-Seq™, 2.9 to 6.1 M dsDCS consensus read pairs were obtained for each library. These dsDCS data were then subjected to mutation analysis.

**Table 2:** Summary of sequencing results and No. of dsDCS read pairs per platform

Sequencer	Sample ID	No. of read pairs sequenced	%Q30^‡^	No. of dsDCS read pairs
HiSeq*	1001	76,865,325	92.19	5006581
	1002	81,271,920	92.17	5219530
	1201	86,124,326	92.41	5747817
	1301	95,845,603	92.62	6155536
NovaSeq	1001	73,009,943	91.65	5145975
	1002	84,338,958	91.15	5209288
	1201	80,358,408	91.05	5230866
	1301	75,894,232	90.80	4949063
NextSeq	1001	61,070,933	93.00	4051603
	1002	62,572,685	92.78	4098438
	1201	61,606,137	93.00	3875417
	1301	66,762,111	92.63	4154968
DNBSeq	1001	90,260,849	93.39	3671187
	1002	50,634,105	92.98	3679493
	1201	76,065,946	94.89	3954380
	1301	77,266,604	94.05	2889421

* Data from a previous study [6], ‡: Percentage of bases of 30 or higher quality score

### Background error frequencies in the four sequencers under Hawk-Seq™ analysis

**Fig. 1:**
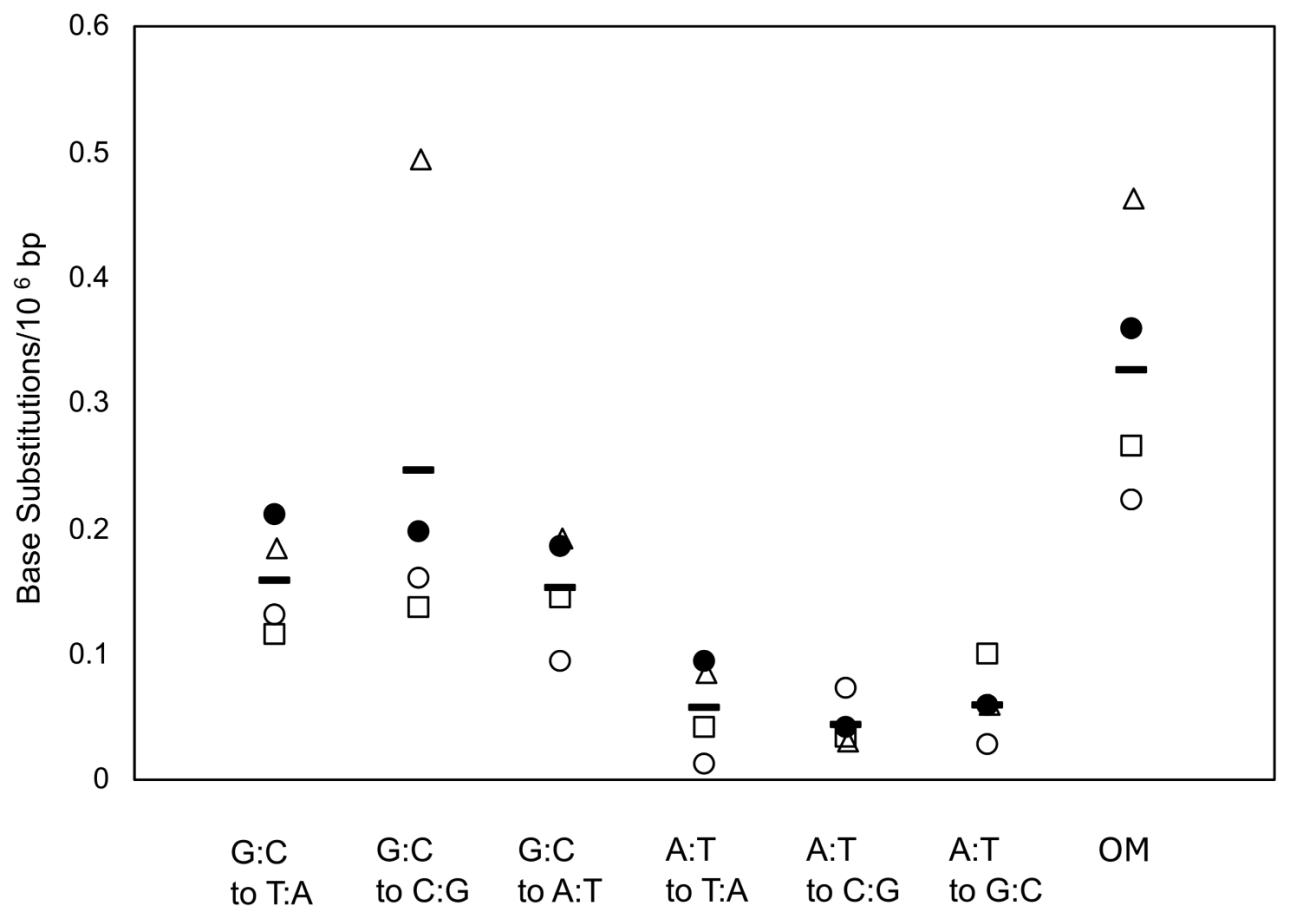
The mean frequencies of 6 types of base substitutions and overall mutations (OM) in the vehicle-treated groups on the four sequencing platforms (*n* = 2). The mutation frequencies per 10^6^ bp of HiSeq (white circles), NovaSeq (black circles), NextSeq (white triangles), and DNBSeq (white rectangles) are displayed. Horizontal bars indicate the mean of the four platforms. HiSeq data were obtained from a previous study [6]. The OM frequency in NextSeq was significantly higher than that of HiSeq (*p* < 0.01 by student’s t-test)

The mean frequencies of overall mutations (OM), 6 types of base substitutions in the vehicle-treated groups (i.e., 1001 and 1002) on each platform, and the mean of the four platforms are shown in Fig. 1. The mean OM frequencies were 0.22 × 10^− 6^, 0.36 × 10^− 6^, 0.46 × 10^− 6^, and 0.26 × 10^− 6^ bp on HiSeq, NovaSeq, NextSeq and DNBSeq, respectively. Illumina platforms were reported to produce errors at the frequency of 10^− 3^ to 10^− 4^ bp [24]. Meanwhile, DNBSeq platforms showed the same level of errors with Illumina HiSeq and NovaSeq determined by *k*-mer analysis [25]. Thus, sequencing errors were dramatically reduced by Hawk-Seq™ in all platforms. Among the four platforms, NovaSeq and NextSeq indicated higher OM frequencies than the highest value (i.e. 0.33 × 10^− 6^) in vehicle control data of HiSeq obtained in our previous study (Supplementary Table 1) [6]. A statistically significant increase was noted in the OM frequency of NextSeq compared to that of HiSeq (*p* < 0.05). The mean values of 6-types of base substitutions among the four platforms were 0.16 × 10^− 6^, 0.25 × 10^− 6^, 0.15 × 10^− 6^, 0.06 × 10^− 6^, 0.04 × 10^− 6^, and 0.06 × 10^− 6^ bp for G:C to T:A, G:C to C:G, G:C to A:T, A:T to T:A, A:T to C:G, and A:T to G:C, respectively. Meanwhile, the coefficient of variations (CVs) of the 6 types of base substitution frequencies among the four platforms were 0.28, 0.67, 0.30, 0.66, 0.44, and 0.47 for G:C to T:A, G:C to C:G, G:C to A:T, A:T to T:A, A:T to C:G, and A:T to G:C, respectively. The relatively higher CV value of G:C to C:G compared to those of other substitution types was mostly due to the value of NextSeq, which indicated a frequency of ca. 0.25 × 10^− 6^ bp higher than the mean of the four platforms. The G:C to C:G frequency in NextSeq was significantly higher than that in HiSeq (*p* < 0.01). The A:T to T:A mutation frequency also indicated a higher CV value (i.e., 0.66) than that of the other substitution types; however, the difference from the mean of the four platforms were 0.05 × 10^− 6^ bp at the largest, which was indicated in HiSeq data.

### Mutation spectra by BP-exposure in the four platforms

**Fig. 2:**
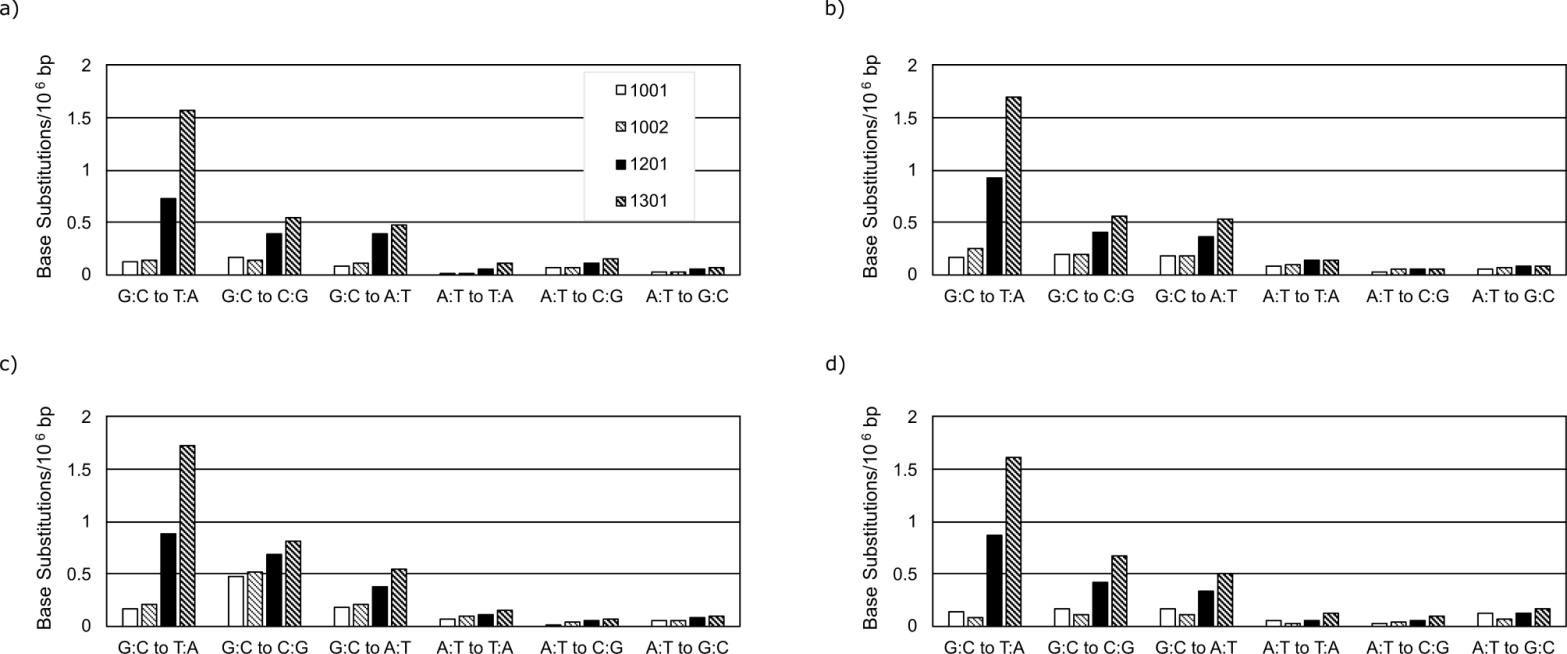
The frequencies of the 6 types of base substitutions in the bone marrow DNA samples of vehicle (1001, 1002) or 150 (1201) and 300 mg/kg (1301) BP-exposed animals. The base substitution frequency in 10^6^ G:C or A:T bp under the analyses using **a**) HiSeq, **b**) NovaSeq, **c**) NextSeq, and **d**) DNBSeq are shown. The data of HiSeq was obtained from a previous study [6]

**Fig. 3:**
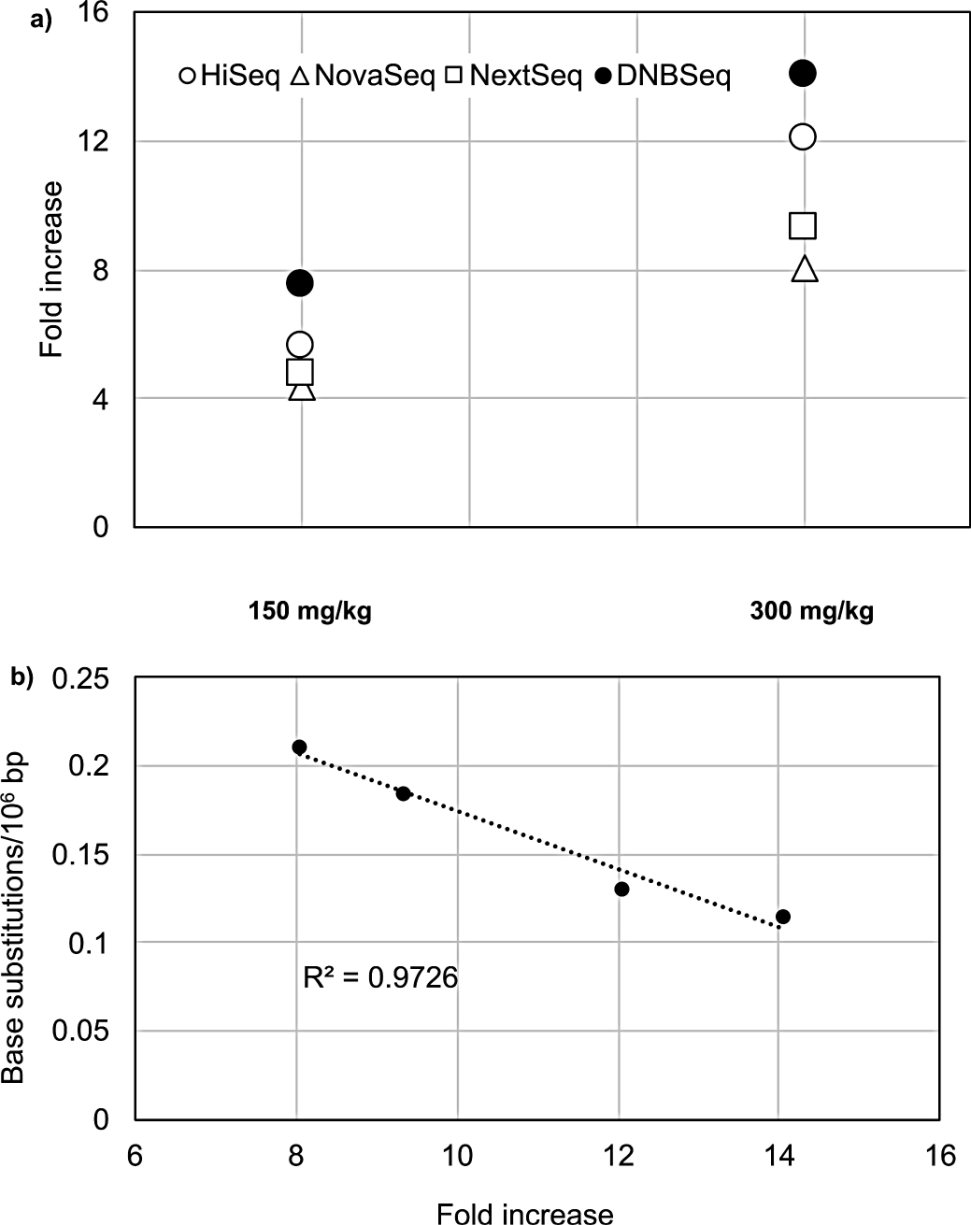
Fold change values on G:C to T:A mutation frequencies in 150 (1201) and 300 mg/kg (1301) BP-exposed animals compared to vehicle controls. **a**) The values of HiSeq (white circles), NovaSeq (white triangles), NextSeq (white rectangles), and DNBSeq (black circles) are displayed. **b**) The correlation between fold change values in G:C to T:A mutation frequencies in 1301 compared to vehicle control and mean G:C to T:A mutation frequencies in 1001 and 1002 samples in the four platforms

Figure 2 shows the frequencies of the 6 types of base substitutions in DNA samples of male *gpt* delta mice exposed to olive oil or 150 and 300 mg/kg BP under the Hawk-Seq™ analysis in the four platforms. On all platforms, a clear dose-dependent increase in G:C to T:A mutation frequencies, which is known as the main substitution pattern induced by BP exposure [6, 26], was observed. Specifically, an increase in at least 4*×* the G:C to T:A mutation frequencies was observed in the 150 mg/kg and 300 mg/kg groups compared to the mean values in each vehicle control and the highest values in historical controls of HiSeq (Fig. 3a and Supplementary Table 2). In control samples, the G:C to T:A mutation frequencies per 10^6^ bp were 0.124, 0.137 for HiSeq, 0.173, 0.248 for NovaSeq, 0.163, 0.206 for NextSeq, and 0.144, 0.085 for DNBSeq. Meanwhile, in samples exposed to 150 mg/kg BP, the G:C to T:A mutation frequencies per 10^6^ bp were 0.73, 0.92, 0.88, and 0.86 on HiSeq, NovaSeq, NextSeq, and DNBSeq, respectively. In samples exposed to 300 mg/kg BP, the G:C to T:A mutation frequencies per 10^6^ bp were 1.69, 1.72, 1.61, and 1.97 on HiSeq, NovaSeq, NextSeq, and DNBSeq, respectively. Thus, all platforms detected an increase in the main base substitution pattern upon exposure to BP. The ranges (i.e. Max. – Min. values) among platforms were 0.163 and 0.150 for controls and 300 mg/kg, respectively. These data suggest that the increase in mutation frequencies caused by BP-exposure were equivalent among platforms. Their differences were caused mostly by variations in background errors.

Figure 3a shows the fold change values of G:C to T:A mutation frequencies compared to vehicle controls in the four platforms. The HiSeq and DNBSeq indicated higher fold change values than NextSeq and NovaSeq especially at 300 mg/kg BP. These values at 300 mg/kg BP were negatively correlated with the mean G:C to T:A mutation frequencies in vehicle controls in the four platforms (Fig. 3b). These data suggest that the background mutation frequency would affect detection sensitivity for mutagen-induced mutations.

### Trinucleotide mutation spectra by BP-exposure in the four platforms

**Fig. 4:**
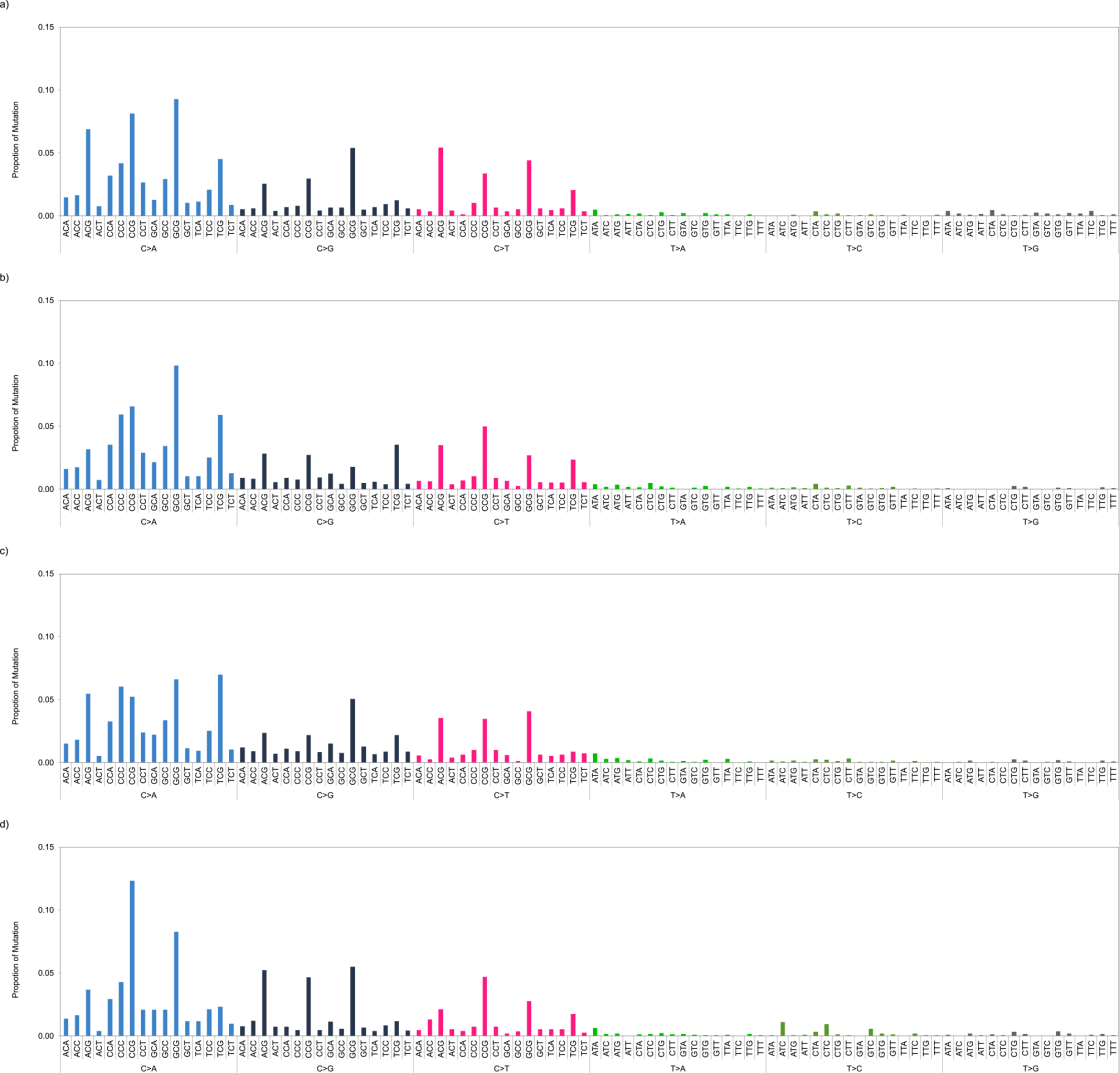
Pattern of 96-dimensional mutational signatures in the liver DNA samples of *gpt* delta mice exposed to 300 mg/kg of BP. The mutation patterns obtained in the analyses using **a**) HiSeq, **b**) NovaSeq, **c**) NextSeq, and **d**) DNBSeq are shown. HiSeq data were obtained from a previous study [6]

Figure 4 shows 96-dimensional trinucleotide mutation patterns (i.e., mutational signatures) in DNA samples of male *gpt* delta mice exposed to 300 mg/kg BP in the four platforms. We calculated the CS values of the mutational signatures between HiSeq and the three other platforms. The CS values were 0.93, 0.95, and 0.92 on NovaSeq, NextSeq, and DNBSeq, respectively. In the study using the duplex sequencing, cosine similarities of the mutation signatures of the same sample between two laboratories ranged from 0.93 to 0.98 [27]. Therefore, our data suggest that all platforms can provide equivalent mutation data that reflect the characteristics of the mutagens.

Furthermore, we calculated CS values between mutational signatures at 300 mg/kg BP in each platform and SBS signatures of the COSMIC database. The relatively higher values (i.e. > 0.5) were noted with SBS4 (tobacco smoking), 24 (aflatoxin), 29 (tobacco chewing), 49 (possible artifact), 87 (thiopurine chemotherapy treatment), 94 (unknown), 95 (possible artifact), and 98 (unknown) (Supplementary Table 3). Among these, SBS4, 24, and 29 also indicated high similarities to BP-induced signature in a previous study [28]. These results suggest that BP-induced signature in each platform reflected characteristics of BP-induced mutations. Additionally, we performed deconstruction of the mutational signature of samples exposed to 300 mg/kg BP in each platform using the deconstructSigs package (Supplementary Table 4). Following this, all sequencers commonly indicated high values in SBS98 (unknown etiology). In addition, high values were indicated in SBS24 (aflatoxin) for HiSeq, SBS16 (unknown) for NovaSeq and NextSeq, and SBS17a (unknown) for DNBSeq. These data suggest that slight differences in the sequence context preferences of mutations would affect decomposition of mutational signatures.

## Discussion

We examined the effects of sequencing platforms on the evaluation of mutagen-induced mutations using the Hawk-Seq™ analysis. The four platforms used in this study detected a dose-dependent increase in mutation frequency in mouse genomic DNA after BP exposure. The fold induction of G:C to T:A mutation frequencies in BP-exposed samples negatively correlated with background error frequencies in each platform, which suggests that differences in platforms might influence mutation detection sensitivity.

While Illumina sequencers create copies of fragmented DNA using bridge PCRs during the sequencing process, DNBSeq creates copies of the DNA ring with rolling circle amplification as DNA nanoballs [16, 29]. The DNBSeq produces data of equal quality using the Illumina platform [16]. In this study, DNBSeq also provided comparable data in terms of both error frequency and mutation signature with the Illumina platforms in the Hawk-Seq™ analysis. Thus, not only Illumina platforms, but also MGI sequencers are applicable to ecNGS-based assays.

Among the four platforms evaluated in this study, NextSeq indicated higher G:C to C:G and overall error frequencies than those of the HiSeq and our historical data. When G to C/C to G calls were counted separately, C to G calls were observed more frequently than G to C calls in the NextSeq analysis (Supplementary Fig. 1). However, we and other investigators have previously reported that G to C is more frequently observed than C to G in ecNGS analysis because of guanine oxidation in the single-stranded regions of fragmented DNA [10, 30–32]. This suggests that incorrect C to G calls would frequently occur, specifically in NextSeq. This is possibly caused by high-quality G bases being overcalled on the Illumina platforms of 2 color chemistry [33]. NovaSeq is also a 2-color chemistry platform; however, NextSeq has been reported to produce errors at higher frequencies than those of the NovaSeq platform [24]. Although this difference might cause false negatives when evaluating mutagens that only induce low G:C to C:G mutations, mutagens that target G:C base pairs generally concurrently induce G:C to T:A and/or G:C to A:T mutations [6, 7, 11]. As NextSeq showed similar values to other platforms in terms of these mutation frequencies, it should be equally sensitive to other platforms.

Differences in other experimental tools could also influence the error frequencies. For example, differences in DNA fragmentation protocols are likely to affect the error frequencies. During DNA fragmentation, several factors can affect the error frequency in NGS analysis, such as differences in methodology [10, 34], solvents [31], and instrument settings [35]. These parameters should be specifically monitored during technical transfers to adequately assess the reproducibility of ecNGS-based assays.

Unlike conventional biological assays, genome sequencing-related devices will continue to progress rapidly; thus, limiting the number of acceptable devices may not be appropriate during standardization [13, 36]. It would be desirable to acknowledge the differences in instruments by utilizing internal controls or setting absolute success criteria for background error frequencies.

## Conclusions

All four sequencing platforms detected mutagen-induced mutations. The background error frequencies can differ depending on the platform used, which might influence detection sensitivity. Therefore, their effects on the mutation analysis should be assessed prior to the experiment. Platforms that provide data with lower error frequencies should be used.

## Electronic supplementary material

Below is the link to the electronic supplementary material.

Supplementary Material 1

## Data Availability

Data will be made available on request.
