# Supplementary material for: Effect of sequencing platforms on the sensitivity of chemical mutation detection using Hawk-Seq™
Source: Genes Environ. 2024 Oct 9;46:20. doi: 10.1186/s41021-024-00313-9 (PMC11462924; doi:10.1186/s41021-024-00313-9)
Supplement: Supplementary file 1 — Supplementary Material 1 [file 41021_2024_313_MOESM1_ESM.docx]

**Effect of sequencing platforms on the sensitivity of chemical mutation detection using Hawk-Seq^TM^**

Sayaka Hosoi^1a^, Takako Hirose^1a^, Shoji Matsumura^1*^, Yuki Otsubo^1^, Kazutoshi Saito^2^, Masaaki Miyazawa^2^, Takayoshi Suzuki^3^, Kenichi Masumura^4^ and Kei-ichi Sugiyama^3^

^1^R&D -Safety Science Research, Kao Corporation, 3-25-14 Tono-machi, Kawasaki-ku, Kawasaki-shi, Kanagawa 210-0821, Japan

^2^R&D -Safety Science Research, Kao Corporation, 2606 Akabane, Ichikai-Machi, Haga-Gun, Tochigi 321-3497, Japan

^3^Division of Genome Safety Science, National Institute of Health Sciences, 3-25-26 Tono-machi, Kawasaki-ku, Kawasaki-shi, Kanagawa, 210-9501, Japan

^4^Division of Risk Assessment, National Institute of Health Sciences, 3-25-26 Tono-machi, Kawasaki-ku, Kawasaki-shi, Kanagawa, 210-9501, Japan

^a^These authors contributed equally to this work.

*To whom correspondence should be addressed. Tel: +81-70-3301-1852; Fax; +81-285-68-7452; Email: matsumura.shouji@kao.com

Supplementary data

a)

b)

c)

d)

e)

**Supplementary Fig. 1**

Frequencies of 12 types of substitutions at G:C bp in the bone marrow DNA samples of vehicle-treated animals (1001 and 1002). a) The mean values of the two animals analyzed using the four sequencing platforms are shown. The values in individual samples using b) HiSeq, c) NovaSeq, d) NextSeq, and e) DNBSeq are shown. The HiSeq data were obtained from a previous study [6].

Supplementary Table 1. Summary of overall mutation frequencies (×10^-6^) in vehicle controls in HiSeq2500 [6]

| Organ | Bone marrow | Bone marrow | Liver | Liver | Kidney | All |
| --- | --- | --- | --- | --- | --- | --- |
| Vehicle | Saline | Olive oil | Saline | Olive oil | Olive oil |  |
| No. of samples | 4 | 4 | 4 | 4 | 4 | 20 |
| Min | 0.162 | 0.206 | 0.262 | 0.287 | 0.179 | 0.162 |
| Max | 0.180 | 0.253 | 0.305 | 0.333 | 0.225 | 0.333 |
| Mean | 0.172 | 0.225 | 0.286 | 0.306 | 0.204 | 0.239 |
| SD | 0.00777 | 0.0201 | 0.0208 | 0.0205 | 0.0204 | 0.0539 |

Supplementary Table 2. Summary of G:C to T:A mutation frequencies (×10^-6^) in vehicle controls in HiSeq2500 [6]

| Organ | Bone marrow | Bone marrow | Liver | Liver | Kidney | All |
| --- | --- | --- | --- | --- | --- | --- |
| Vehicle | Saline | Olive oil | Saline | Olive oil | Olive oil |  |
| No. of samples | 4 | 4 | 4 | 4 | 4 | 20 |
| Min | 0.0861 | 0.0901 | 0.108 | 0.106 | 0.0800 | 0.0800 |
| Max | 0.121 | 0.137 | 0.154 | 0.165 | 0.127 | 0.165 |
| Mean | 0.110 | 0.115 | 0.132 | 0.128 | 0.114 | 0.120 |
| SD | 0.0165 | 0.0202 | 0.0185 | 0.0257 | 0.0229 | 0.0207 |

Supplementary Table 3. Cosine similarities between mutational signatures of samples exposed to 300 mg/kg of BP and COSMIC SBS signatures

|  | SBS4 | SBS24 | SBS29 | SBS49 | SBS87 | SBS94 | SBS95 | SBS98 |
| --- | --- | --- | --- | --- | --- | --- | --- | --- |
| HiSeq | 0.520 | 0.548 | 0.502 | 0.494 | 0.472 | 0.476 | 0.458 | 0.609 |
| NovaSeq | 0.603 | 0.612 | 0.565 | 0.459 | 0.508 | 0.557 | 0.533 | 0.617 |
| NextSeq | 0.605 | 0.592 | 0.567 | 0.408 | 0.437 | 0.569 | 0.529 | 0.584 |
| DNBSEQ | 0.496 | 0.491 | 0.449 | 0.645 | 0.471 | 0.432 | 0.436 | 0.697 |
| Proposed etiology | Tobacco smoking | Aflatoxin | Tobacco chewing | Possible artifact | Thiopurine chemotherapy treatment | Unknown | Possible artifact | Unknown |

Supplementary Table 4. SBS compositions of mutational signatures in BP-exposed samples in each platform determined by deconstructSigs

|  | SBS1 | SBS4 | SBS16 | SBS17a | SBS24 | SBS45 | SBS48 | SBS49 | SBS98 |
| --- | --- | --- | --- | --- | --- | --- | --- | --- | --- |
| HiSeq | 0.104 | 0 | 0 | 0 | 0.340 | 0 | 0 | 0.0874 | 0.355 |
| NovaSeq | 0.0635 | 0 | 0.364 | 0 | 0 | 0.0927 | 0 | 0 | 0.336 |
| NextSeq | 0.0669 | 0.109 | 0.286 | 0 | 0 | 0.0700 | 0.0751 | 0 | 0.316 |
| DNBSEQ | 0 | 0 | 0 | 0.282 | 0 | 0 | 0 | 0.164 | 0.498 |
| Proposed etiology | Deamination of 5-meC | Tobacco smoking | Unknown | Unknown | Aflatoxin | SA* | SA | SA | Unknown |

*: Possible sequencing artifact
